# Multiomics Analysis of Liver Molecular Dysregulation Leading to Nonviral-Related Hepatocellular Carcinoma Development

**DOI:** 10.1021/acs.jproteome.4c00729

**Published:** 2025-02-21

**Authors:** Hikaru Nakahara, Atsushi Ono, C. Nelson Hayes, Yuki Shirane, Ryoichi Miura, Yasutoshi Fujii, Yosuke Tamura, Shinsuke Uchikawa, Hatsue Fujino, Takashi Nakahara, Eisuke Murakami, Masami Yamauchi, Tomokazu Kawaoka, Daiki Miki, Masataka Tsuge, Tsuyoshi Kobayashi, Hideki Ohdan, Koji Arihiro, Shiro Oka

**Affiliations:** † Department of Gastroenterology, Graduate School of Biomedical & Health Sciences, Hiroshima University, Hiroshima 734-8551, Japan; ‡ Department of Clinical and Molecular Genetics, Hiroshima University, Hiroshima 734-8551, Japan; § Department of Clinical Oncology, Graduate School of Biomedical and Health Sciences, Hiroshima University, Hiroshima 734-8551, Japan; ∥ Hiroshima Prefectural Hospital Gastroenterology & Hepatology, Hiroshima 734-8530, Japan; ⊥ Department of Clinical Oncology, Hiroshima Prefectural Hospital, Hiroshima 734-8530, Japan; # Liver center, Hiroshima University Hospital, Hiroshima 734-8551, Japan; ∇ Department of Gastroenterological and Transplant Surgery, Graduate School of Biomedical and Health Sciences, Hiroshima University, Hiroshima 734-8551, Japan; ○ Department of Anatomical Pathology, Hiroshima University Hospital, Hiroshima 734-8551, Japan

**Keywords:** NAD metabolism, acylcarnitine, fatty acid, NAFLD, MOVICS

## Abstract

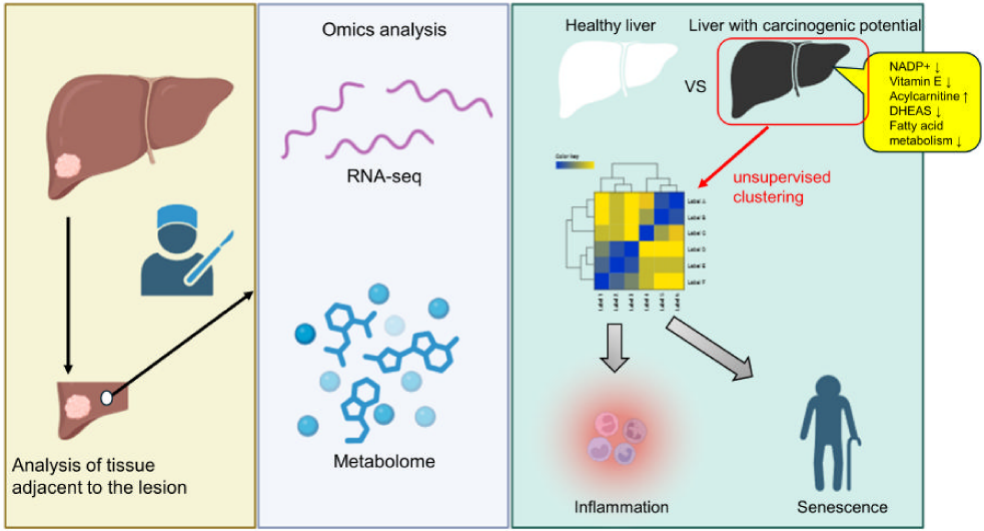

Chronic liver diseases exhibit diverse backgrounds, and it is believed that numerous factors contribute to progression to cancer. To achieve effective prevention of nonviral hepatocellular carcinoma, it is imperative to identify fundamental molecular abnormalities at the patient level. Utilizing cancer-adjacent liver tissues obtained from hepatocellular carcinoma patients (chronic liver disease), we conducted RNA-Seq and metabolome analyses. In the chronic liver disease cohort, upregulation of inflammation-associated signals was observed, concomitant with accumulation of acylcarnitine and fatty acid and depletion of NADP+, gamma-tocopherol, and dehydroisoandrosterone-3-sulfate-1 (DHEAS). To minimize heterogeneity, we performed multiomics clustering, successfully categorizing the chronic liver disease cases into two distinct subtypes. Subtype 1 demonstrated elevated inflammatory levels, whereas Subtype 2 included a disproportionately high proportion of elderly cases. Furthermore, RNA-Seq analysis revealed upregulation of inflammatory signals in Subtype 1, while both subtypes exhibited downregulation of fatty acid metabolism. Metabolome analysis indicated a tendency of increased acylcarnitine levels in Subtype 1 and augmented fatty acid accumulation in Subtype 2. Validation of differentially expressed genes using the Gene Expression Omnibus (GEO) data set revealed the potential for amelioration through supplementation with antioxidants such as epigallocatechin gallate (EGCG).

## 1 Introduction

Primary liver cancer was the sixth most commonly diagnosed cancer and the third leading cause of cancer death worldwide in 2020.^1^ Recently, the incidence of hepatocellular carcinoma (HCC) associated with nonviral chronic liver disease (CLD) has gradually increased to approximately 15–25%.^2−5^ Frequencies of liver diseases associated with cases of nonviral HCC have been reported as follows: alcoholic liver disease (ALD) (43–51%), so-called cryptogenic liver disease of unknown etiology (18–35%), and nonalcoholic fatty liver disease (NAFLD) (13–28%).^4,6−8^

Tokushige et al. reported that the most common etiology among nonviral HCC patients under 80 years old was ALD, whereas those aged 80 years or older were cryptogenic. The prevalence of obesity, diabetes mellitus (DM), and liver cirrhosis in the 80 years or older group of cryptogenic HCC patients were significantly lower than those of younger patients.^8^ Several studies have reported risk factors for hepatocarcinogenesis in patients with nonviral hepatitis. Male gender,^9^ older age,^9^ past obesity,^9^ DM,^9^ abnormal levels of transaminases^9^ and tobacco consumption^9^ were associated with HCC in patients with nonviral cirrhosis and many risk factors for NAFLD-HCC have also been reported.^10^

In recent years, some studies have been reported to analyze the prognostic signatures and subtypes associated with NAFLD.^11,12^ However, few studies have investigated the molecular abnormalities directly involved in the development of HCC. In addition, because many nonviral HCC patients have multiple risk factors, identifying the primary cause in individual cases remains challenging, complicating efforts to develop effective treatments. To address this, we hypothesized that an unsupervised clustering approach that does not rely on clinical information could classify molecularly heterogeneous chronic liver diseases into distinct subgroups and reveal individualized treatment targets.

In this study, we analyzed noncancerous liver tissue adjacent to HCC lesions from patients with nonviral chronic liver disease. Through multiomics analysis of transcriptomic and metabolomic data, we aimed to uncover molecular mechanisms underlying HCC development and identify novel targets for chemoprevention.

## 2 Materials and Methods

### 2.1 Patients

We included all cases that underwent hepatic resection at our institution between March 2009 and October 2019, met the exclusion criteria described below, had postsurgical follow-up at our institution, provided written informed consent, and had adequately preserved frozen tissue samples.

Exclusion criteria were as follows: (i) patients with a history of systemic drug therapy prior to surgery, (ii) patients considered to have underlying involvement of hepatitis C, hepatitis B, autoimmune hepatitis (AIH), or primary biliary cholangitis (PBC). Specifically, patients positive for HBsAg, anti-HCV antibodies, antinuclear antibodies (ANA), or antimitochondrial M2 antibodies were excluded. Patients negative for ANA but meeting the diagnostic criteria for AIH outlined in the 2021 Japanese Ministry of Health, Labour and Welfare guidelines were also excluded. In brief, AIH is diagnosed, including atypical cases, if one or more of the following criteria (1–4) are met along with criterion 5:1) positive for ANA or antismooth muscle antibodies (ASMA), 2) elevated IgG levels (>1.1 times the upper limit of normal), 3) histological findings of interface hepatitis or plasma cell infiltration, 4) marked response to corticosteroids, and 5) exclusion of liver damage caused by other factors. After excluding patients without pathological inflammation or fibrosis to minimize the impact of genetic issues, 56 patients remained in the CLD group. An additional 13 patients who had undergone hepatectomy for hemangioma or metastatic tumor were enrolled as a control group. The clinical information measured immediately before surgery is shown in Table 1. Fibrosis in the resected cancer-adjacent liver was assessed using the New Inuyama Classification.^13^ All patients and control subjects provided written informed consent for study participation.

**Table 1:** Clinical Information on Control versus CLD Groups and CLDS1 versus CLDS2^a^

	Control vs CLD			CLDS1 vs CLDS2		
	Control	CLD	*P* value	CLDS1	CLDS2	*P* value
Sex						
Male (%)	6 (46.2)	48 (85.7)	0.0049	27 (87.1)	15 (78.9)	0.49
Female (%)	7 (53.8)	8 (14.3)		4 (12.9)	4 (21.1)	
Age, median (IQR)	62.3 (43.5–68.5)	74.5 (69.2–79.7)	<0.0001	72.6 (68.5–75.5)	79.2 (72.3–82.0)	0.002
BMI, median (IQR)	23.3 (19.6–25)	23.5 (21.8–25.6)	0.23	23.8 (21.9–25.6)	22.3 (20.4–27.3)	0.83
Fibrosis						
F0 (%)	13 (100)	0 (0)	<0.0001	0 (0)	0 (0)	0.77
F1–2 (%)	0 (0)	34 (60.7)		18 (58.1)	12 (63.2)	
F3–4 (%)	0 (0)	22 (39.3)		13 (41.9)	7 (36.8)	
*NAS* (n = 68)						
Steatosis						
0 (%)	9 (69.2)	37 (67.3)	0.22	20 (64.5)	14 (73.7)	0.55
1 (%)	3 (23.1)	18 (32.7)		11 (35.5)	5 (26.3)	
2 (%)	0 (0)	0 (0)		0 (0)	0 (0)	
3 (%)	1 (7.7)	0 (0)		0 (0)	0 (0)	
Hepatocyte ballooning						
0 (%)	9 (69.2)	35 (63.6)	1	20 (64.5)	12 (63.2)	0.19
1 (%)	2 (15.4)	12 (21.8)		8 (25.8)	2 (10.5)	
2 (%)	2 (15.4)	8 (14.5)		3 (9.7)	5 (26.3)	
Lobular inflammation						
0 (%)	12 (92.3)	1 (1.8)	<0.0001	0 (0)	0 (0)	0.0006
1 (%)	1 (7.7)	25 (45.5)		9 (29.0)	14 (73.7)	
2 (%)	0 (0)	26 (47.3)		21 (67.7)	3 (15.8)	
3 (%)	0 (0)	3 (5.5)		1 (3.2)	2 (10.5)	
Type 2 diabetes						
No (%)	8 (61.5)	20 (35.7)	0.12	10 (32.3)	9 (47.4)	0.37
Yes (%)	5 (38.5)	36 (64.3)		21 (67.7)	10 (52.6)	
Hyperlipidemia						
No (%)	11 (84.6)	38 (67.9)	0.32	22 (71.0)	13 (68.4)	1
Yes (%)	2 (15.4)	18 (32.1)		9 (29.0)	6 (21.6)	
Steatosis						
No (%)	9 (69.2)	35 (62.5)	0.55	18 (58.1)	14 (73.7)	0.51
Mild (%)	3 (23.1)	17 (30.4)		9 (29.0)	5 (26.3)	
Moderate (%)	0 (0)	3 (5.4)		3 (9.7)	0 (0)	
Severe (%)	1 (7.7)	1 (1.8)		1 (3.2)	0 (0)	
Alcohol consumption						
Never (%)	3 (23.1)	19 (33.9)	0.079	7 (22.6)	8 (42.1)	0.33
Social (%)	7 (53.8)	12 (21.4)		7 (22.6)	4 (21.1)	
Heavy (%)	3 (23.1)	25 (44.6)		17 (54.8)	7 (36.8)	
AST, median (IQR)	19 (15–25)	30 (25–35)	0.0008	30 (26–35)	25 (20–34)	0.11
ALT, median (IQR)	16 (11–32)	27 (19–32)	0.072	28 (19–33)	26 (19–29)	0.23
γGTP, median (IQR)	27 (19–47.5)	59 (37–92.75)	0.0007	62 (41–92)	58 (36–95)	0.58
Alb, median (IQR)	4.4 (4.1–4.6)	3.9 (3.6–4.2)	0.0026	3.9 (3.6–4.2)	3.8 (3.5–4.3)	0.94

a Abbreviations: BMI, body mass index; NAS, NAFLD activity score; AST, aspartate aminotransferase; ALT, alanine aminotransferase; γGTP, gamma-glutamyl transpeptidase; Alb, albumin.

### 2.2 NAFLD Activity Score

NAFLD Activity Score (NAS), which scores the degree of steatosis, hepatocyte ballooning, and lobular inflammation to evaluate histological activity, was evaluated using hematoxylin and eosin staining (HE staining) of liver surgical specimens. However, pathological specimens could not be obtained in one of the 69 cases and could not be evaluated.

### 2.3 Samples

Adjacent liver tissues were collected at the sites of surgical resection for HCC, metastatic liver tumor, or hemangioma. Collected tissue was stored at −80 °C as fresh frozen.

#### 2.3.1 Total RNA-Seq

A Petri dish was placed on dry ice, and tissue samples were sectioned into pieces no larger than 5 mm in any dimension. These pieces were placed into screw-cap tubes containing RNAlater and transported frozen to the Bioengineering Laboratory (Kanagawa, Japan), where RNA extraction and sequencing were performed. Total RNA-Seq was performed using the DNBSEQ-G400 system. 8,820 million paired reads were obtained. Total RNA was extracted from fresh frozen liver tissue using QIAGEN’s RNeasy kits in accordance with established protocols. Extracted RNA was treated with Zymo Research’s RNA Clean & Concentrator-5 with DNase I. The MGIEasy RNA Directional Library Prep Set and MGISP 100 were used to create DNB. Adapters from the MGIEasy DNA Adapters 96 (Plate) Kit were also used. Qubit 30 Fluorometer and dsDNA HS Assay Kit (Thermo Fisher Scientific) were used for library quantification, while the Fragment Analyzer and dsDNA 915 Reagent Kit (Advanced Analytical Technologies) and the Agilent 2100 Bioanalyzer and High Sensitivity DNA Kit (Agilent Technologies) were used for quality control. Sequencing was conducted using paired-end reads of 150 bp. Each process was performed according to the manufacturer’s protocol. Quantification, quality control results, and sequencing total reads are presented in Supporting Information (Table S1).

#### 2.3.2 Metabolome Analysis

Metabolome analysis was conducted using CE-TOFMS and LC-TOFMS at Advanced Scan Plus (HMT) in Yamagata, Japan. Thirty mg of tissue for CE-TOFMS and 50 mg of tissue for LC-TOFMS were sectioned on a Petri dish placed on dry ice. The samples were placed in HMT-specified tubes and shipped frozen to HMT. At HMT, the tissue underwent pulverization, ultrafiltration, and solid-phase extraction prior to mass spectrometry analysis. Information on the samples shipped and dilutions is provided in Table S2. Detailed protocols for CE-TOFMS and LC-TOFMS are also provided in Text S1.

### 2.4 Analysis Workflow

To compare the differences between control and CLD, statistical analyses were performed using RNA-Seq and metabolomics methods. Subsequently, to reduce heterogeneity in CLD, integrated clustering was performed using multiple omics data (Figure S1). Data were not available for metabolomic analysis as 6 out of 56 CLDs and 1 out of 13 controls were ineligible for Dual Scan. Clustering was performed using 50 CLD cases for which all RNA-Seq and metabolome data were available. Then, differences with respect to controls were compared for each of the subtypes obtained.

### 2.5 Transcriptome Analysis

Sequencing reads were preprocessed using fastp v0.20. RSEM was used for mapping and quantification to the human genome GRCh38.p13. STAR was selected as the parameter for mapping. The GRCh38.101 gtf file obtained from Ensembl was used for annotation. Differential gene expression analysis was performed with the R package DESeq2, as it employs a robust statistical model that accounts for variability in RNA-seq data across biological replicates. The Wald test implemented in DESeq2 was used for statistical analysis to estimate the significance of differential expression, as it is specifically designed to handle the negative binomial distribution of RNA-seq counts. For the downstream analysis, we used data normalized using the median of ratios method of DESeq2 and variance stabilizing transformation.

### 2.6 Metabolome Analysis

The Basic Scan provided data on 374 metabolites for 69 patients, and the Dual Scan provided data on 223 metabolites for 62 patients. 62 patients for whom both Basic Scan and Dual Scan data were available were used in the analysis. We used the open source MetaboAnalyst 5.0 server (https://www.metaboanalyst.ca) to preprocess the data. Features with more than 50% missing values were removed, and the remaining missing values were complemented by Bayesian principal component analysis. Pairwise Wilcoxon test with Bonferroni correction was used for statistical analysis because it is a nonparametric test suitable for comparing metabolite levels between groups, especially when the data distribution is not normal. The Bonferroni correction was applied to control for multiple testing and to reduce false positives given the large number of metabolites analyzed. The R package ggplot2 was used for plotting. For downstream analysis, we used data processed with MetaboAnalyst 5.0 server under the conditions described above. The relationships between metabolites and metabolic pathways were manually curated.

### 2.7 Principal Component Analysis

Principal Component Analysis (PCA) was performed to examine the distribution of the data. The R package ggfortify v0.4.16 was used for analysis and plotting.

### 2.8 mixOmics

We used the R package mixOmics v6.16.3,^14^ a supervised method based on multiblock sparse partial least-squares discriminant analysis, to integrate the transcriptome and metabolome for biomarker discovery. For clustering, 5000 genes with high median absolute deviation (MAD) were extracted, and all metabolites were used.

### 2.9 Multiomics Clustering

We used the R package Multi-Omics Integration and VIsualization in Cancer Subtyping (MOVICS)^15^ for the multiomics clustering. For clustering, 1000 genes with high MAD were extracted, and all metabolites were used. The getClustNum() function was used to calculate the optimal number of clusters between 2 and 8, and the recommended *k* = 2 was selected (Figure S2A). IntNMF and iClusterBayes were used for clustering (Figure S2B).

### 2.10 Prognostic Liver Signature Analysis

Prognostic liver signature (PLS) was classified using nearest template prediction (NTP) implemented in the R package CMScaller v2.0.1. The genes to be used were determined based on the literature.^12,16^ The significance level was set at FDR < 0.05, and those not outside of this threshold were set as not available (NA).

### 2.11 Gene Set Enrichment Analysis

Enrichment analysis was performed using Gene Set Enrichment Analysis (GSEA) software (https://www.gsea-msigdb.org/) with the Hallmark gene set.

### 2.12 Annotation of Genes with Differential Expression

We used the R package clusterProfiler v4.0.5 to annotate genes that were differentially expressed in each group.

### 2.13 Relapse-Free Survival Analysis

Relapse-Free Survival (RFS) analysis was performed using cases that had been cured by surgical resection at the time the omics sample had been taken. The two groups had 15 and 11 eligible cases, respectively, and were analyzed using the survival package and depicted using the survminer package v0.4.9 in R.

### 2.14 Survival Rate Analysis

A postsurgical survival analysis was performed on the two groups obtained by MOVICS, using the survival package and depicted using the survminer package in R. The two groups consisted of 31 and 19 cases, respectively, with a median follow-up of 2089 days.

### 2.15 Cox Proportional Hazards Regression Analysis

Cox proportional hazards regression analysis was performed using the lifelines v0.27.8 package in Python. In multivariate analysis, variables that satisfied *p* < 0.05 in univariate analysis were used.

### 2.16 Cell Type Enrichment Analysis

Cell type enrichment analysis was performed using TPM-corrected gene expression data obtained from RSEM. The R package xCell v1.1.0^17^ was used for analysis and plotted using ggplot2.

### 2.17 Signatures Validated on Gene Expression Omnibus Data Set

We examined gene signatures using the publicly available Gene Expression Omnibus (GEO, https://www.ncbi.nlm.nih.gov/geo) data set. The effects of aging were investigated using GSE183915 and GSE108978, while the effects of substances causing liver damage were investigated using GSE115473, GSE188604 and GSE119953. The effects of various nutrients on animal models of chronic liver injury were also examined using GSE77964, GSE35961, GSE51432, GSE93819, GSE186165, GSE94593 and GSE137840.

## 3 Results

### 3.1 Molecular Comparison between Control and CLD Groups

**Figure 1:**
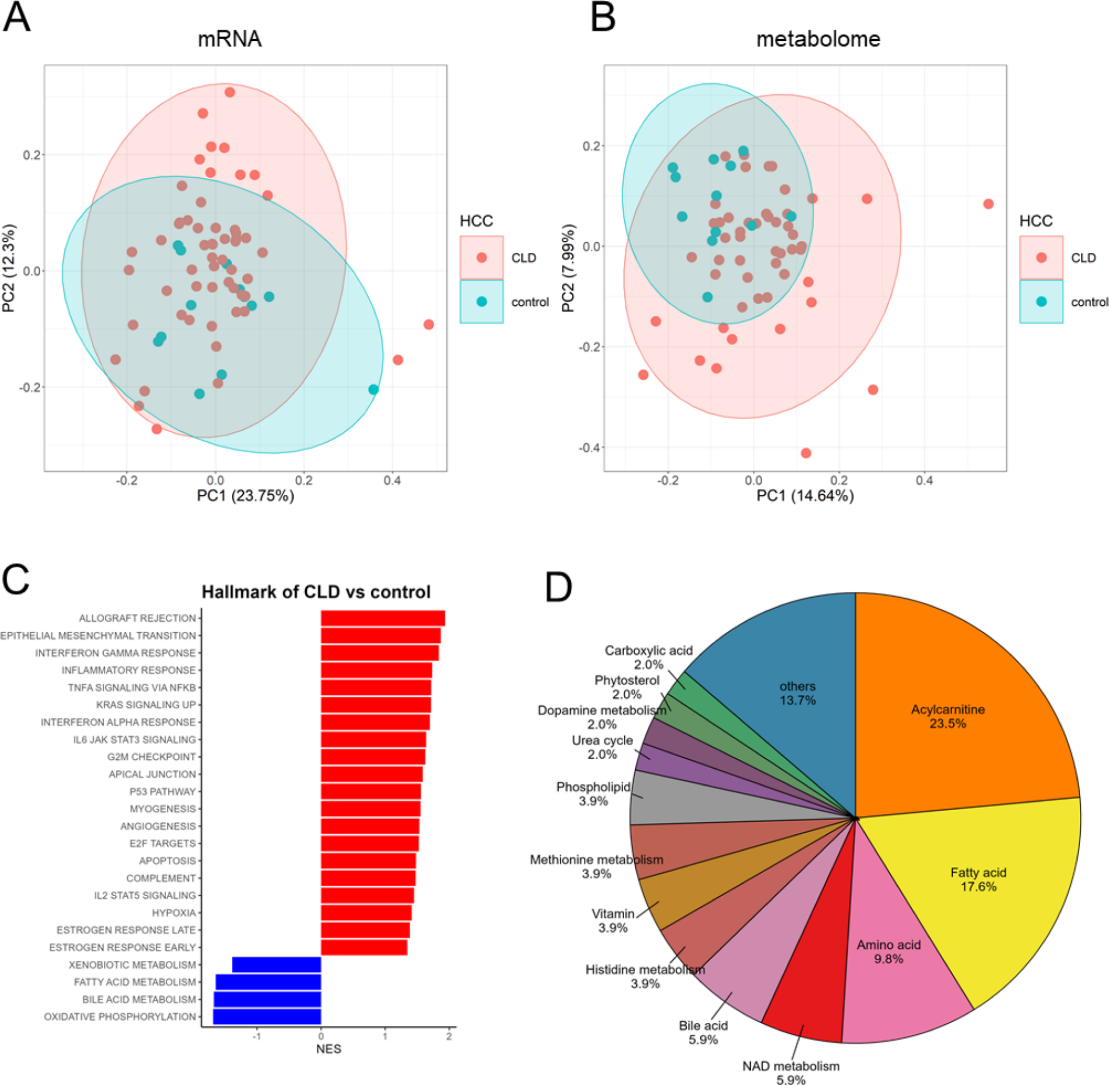
Statistical analysis of control vs CLD. (A) PCA plots using RNA-Seq data. (B) PCA plots using metabolomics data. (C) GSEA analysis comparing CLD and control groups; red represents a positive correlation while blue represents a negative correlation at FDR < 0.05. (D) Percentage of each type of metabolite with significant differences.

To investigate molecular aberrations in CLD, RNA-Seq and metabolome data were compared with the control group. GSEA and PCA were performed using RNA-Seq. GSEA revealed 20 positively enriched pathways in CLD; e.g., inflammation, epithelial-mesenchymal transition; EMT and 4 negatively enriched pathways, including fatty acid metabolism (Figure 1C). Further PCA revealed that subpopulations within the CLD group were closer to or farther from the control group (Figure 1A). The results of the differential expression analysis using DESeq2 are shown in Table S3.

PCA using metabolome data showed that the CLD group was more widely distributed than the control group (Figure 1B). Factor loadings showed a higher contribution from long-chain fatty acids in PC1 and acylcarnitines in PC2. Differential Metabolites (DEM) with an adjusted *p* value <0.05 were extracted using MetaboAnalyst 5.0, and 48 metabolites were found to be higher and 3 metabolites were found to be lower in the CLD group. The accumulated metabolites contained 12 acylcarnitines and 8 fatty acids, while the decreased metabolites contained NADP+ gamma-tocopherol and dehydroisoandrosterone-3-sulfate-1 (DHEAS). The results of the differential analysis are shown in Table S4, and the types of metabolites having significant differences are shown in the pie chart (Figure 1D). We further integrated the metabolome and transcriptome using mixOmics. This revealed transcripts and metabolites that characterize CLD. (Figure S3).

### 3.2 Comparison of Metabolomes with Different Backgrounds

**Figure 2:**
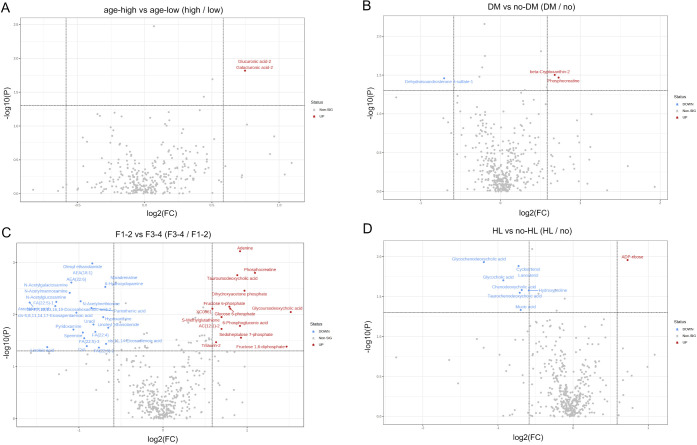
Comparison of metabolomes with different backgrounds. The patients were divided into two groups based on differences in clinical background, and a volcano plot was drawn. (A) High-age vs low-age divided by median. (B) With or without DM. (C) Fibrosis stage 1–2 vs 3–4 (F1–2 vs F3–4). (D) With or without HL.

To investigate the contribution of different background liver types to the metabolome, volcano plots were drawn for two groups according to age, DM, fibrosis, hyperlipidemia (HL) and fatty liver using MetaboAnalyst 5.0 (Figure 2). Each parameter was divided into two groups as follows: above or below median age; fibrosis F1–F2 or F3–F4; and presence or absence of DM and HL. The numbers of metabolites for which a significant difference of *p* < 0.05 were identified were 1 for age, 3 for DM, 8 for HL and 35 for fibrosis (Table S5). As fibrosis progresses, long-chain fatty acids, omega-3, and omega-6 fatty acids were found to decrease, suggesting a relationship with the disappearance of fatty liver called “burned-out nonalcoholic steatohepatitis (NASH)″. However, the effect of ω3 fatty acids on liver fibrosis remains controversial.^18^ Moreover, the accumulation of the glycolytic intermediates Glucose 6-phosphate and Fructose 6-phosphate, and Sedoheptulose 7-phosphate and 6-Phosphogluconic acid in the pentose phosphate pathway suggests that these metabolic interactions may be impaired.

### 3.3 Clustering Multiomics Data with MOVICS

**Figure 3:**
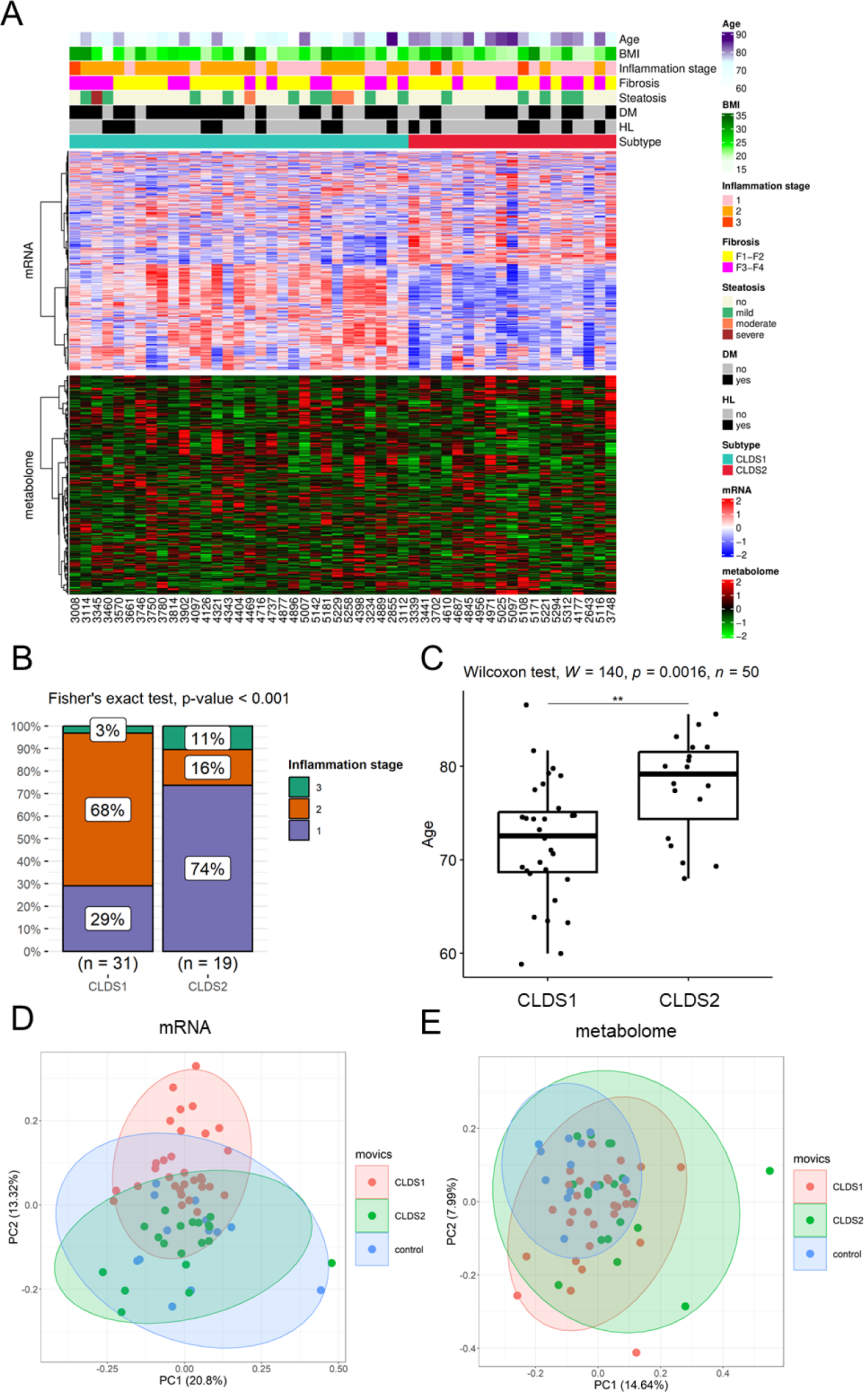
Subtype classification of CLDs. (A) Clustering multiomics data with MOVICS. Clinical information, CLDS1 and CLDS2 classification, and transcriptome and metabolome expression data in 50 patients are shown. The transcriptome data includes the 1000 genes used for clustering, and the metabolome includes the expression levels of 402 metabolites. (B) Comparison of the distributions of inflammation stage between CLDS1 and CLDS2 by Fisher’s exact test. (C) Comparison of age between CLDS1 and CLDS2 by Wilcoxon test. (D) PCA plot with RNA-Seq colored by MOVICS clustering. (E) PCA plot with metabolome data colored by MOVICS clustering.

To investigate molecular differences, MOVICS analysis was performed based on the RNA-Seq and Metabolome analysis on surrounding liver tissue in 50 CLD patients. Clustering using 1000 genes extracted by MAD from the RNA-Seq data and all metabolites (402) revealed that the cases could be classified into two distinct groups: CLDS1 (31 cases with characteristics of high histological stages of inflammation) and CLDS2 (19 cases with older age characteristics) (Figure 3A–C). There were no significant differences in fibrosis stage and history of DM nor HL in the two groups (Table 1). PCA in 62 patients, including the control group, showed relatively clear separation between CLDS1 and CLDS2 in the transcriptome but not in the metabolome results (Figure 3D,E). In the transcriptome, CLDS2 has a high overlap with the control group, whereas CLDS1 is located farther away from the control.

We also investigated the relationship between our clustering results and the PLS, which predicts the prognosis of hepatocellular carcinoma using 186 genes,^16^ and NAFLD-related PLS (PLS-NAFLD), which uses 133 genes^12^ (Figure S4A). Poor prognosis of PLS was significantly more common in CLDS1, whereas high-risk of PLS-NAFLD tended to be more common in CLDS1, but the difference was not significant (Figure S4B).

### 3.4 Differential Analysis

**Figure 4:**
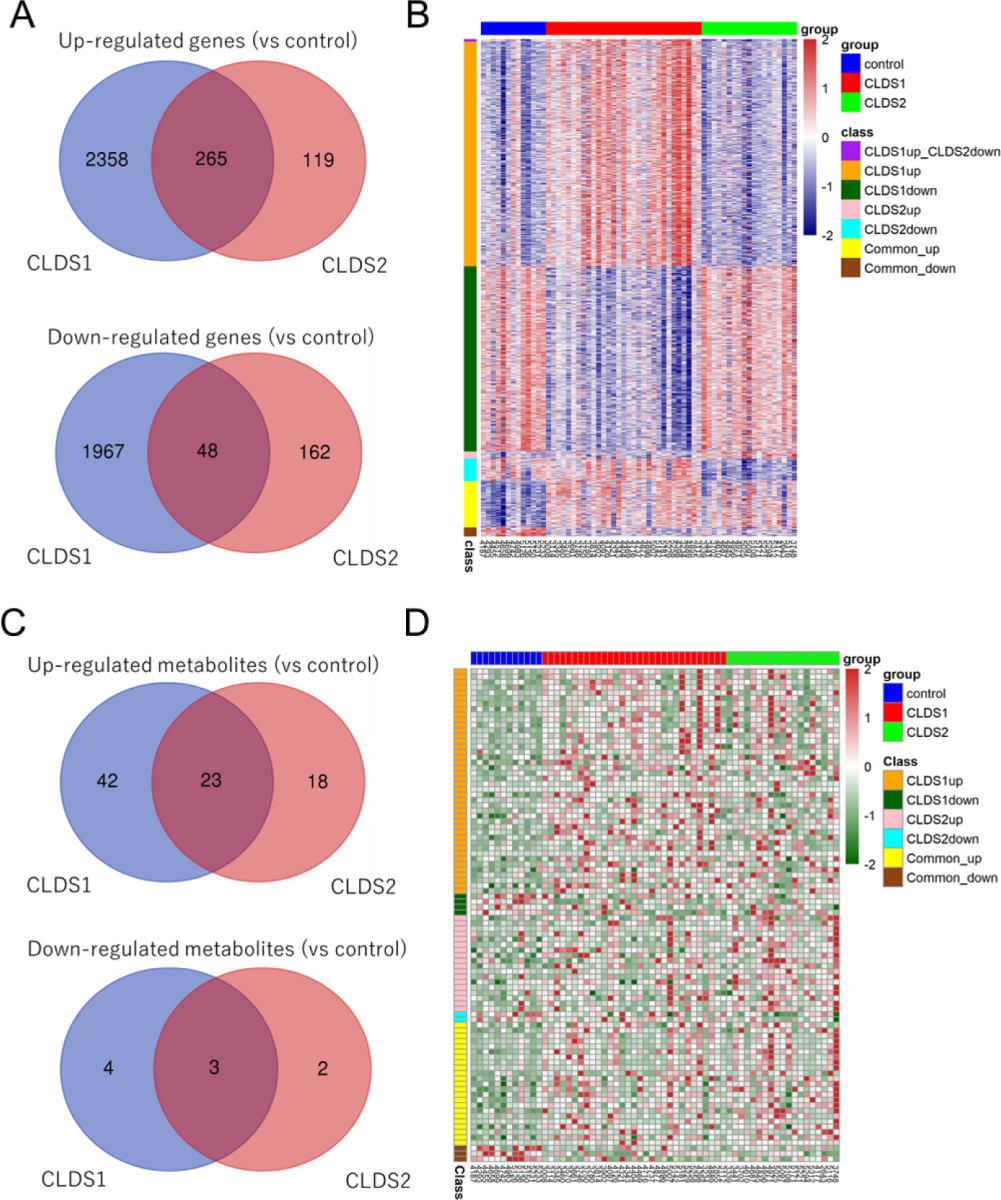
Differential analysis of genes and metabolites. (A) Venn diagram showing genes commonly up- or down-regulated in CLDS1 and CLDS2. (B) Heatmap of genes shown in (A). (C) Venn diagram showing metabolites commonly increased or decreased in CLDS1 and CLDS2. (D) Heatmap of metabolites shown in C.

Differentially Expressed Genes (DEGs) satisfying adjusted *p* value <0.05 were extracted using DESeq2 when CLDS1 and CLDS2 were compared to a control group consisting of 13 patients with no history of HCC. In CLDS1, 2623 genes were upregulated, and 2015 genes were downregulated. On the other hand, 384 genes were upregulated, and 210 genes were downregulated in CLDS2. 265 and 48 genes were commonly upregulated and downregulated, respectively (Figure 4A). All genes with significant differences are shown in the heatmap (Figure 4B).

DEM with adjusted *p* value <0.05 were extracted using the pairwise Wilcoxon test. 65 metabolites were accumulated, and 7 were depleted in CLDS1. In CLDS2, 41 metabolites were accumulated, and 5 metabolites were decreased. Accumulation of 23 and decrease of 3 metabolites were commonly observed in the CLDS1 and CLDS2 groups (Figure 4C). All metabolites with significant differences are shown in the heatmap (Figure 4D).

### 3.5 GSEA and Metabolic Change

**Figure 5:**
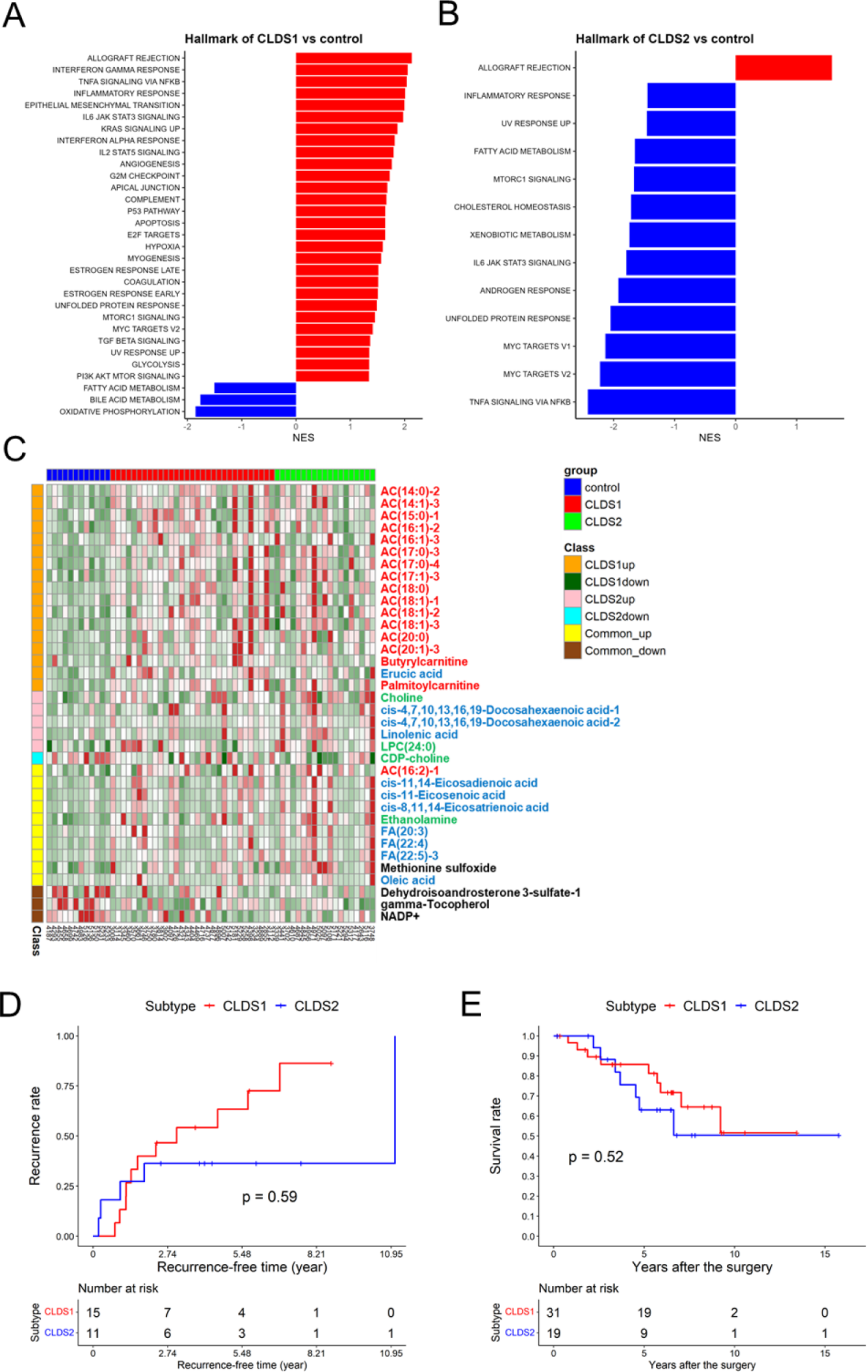
Characteristics of the two subtypes. (A) Pathways that were enriched in CLDS1 vs controls. (B) Pathways that were enriched in CLDS2 vs controls. The significance level is FDR < 0.05. (C) Heatmap of differential metabolites; red font: acylcarnitine, blue font; fatty acid, green font: phosphatidylcholine synthesis related metabolites, black font: others. (D) Kaplan–Meier recurrence-free survival comparison analysis of CLDS1 vs CLDS2. (E) Kaplan–Meier survival rate analysis of CLDS1 vs CLDS2.

GSEA was used to identify enriched gene sets in a particular group. Twenty-eight pathway-related gene sets including carcinogenesis-associated pathways such as inflammation and EMT were positively enriched and 3 pathways including metabolism of fatty acids were negatively enriched in the CLDS1 group compared to the control group (Figure 5A).

On the other hand, pathways involved in fatty acid metabolism were similarly negatively enriched in CLDS2, but in contrast to CLDS1, pathways related to inflammation were also negatively enriched (Figure 5B). All GSEA data are available in Table S6. These differences suggest that the two subtypes may induce CLD by different mechanisms.

Metabolites of particular interest are shown in a heatmap in Figure 5C. In the metabolites that were only accumulated in CLDS1, 16 were long-chain acylcarnitines. One long-chain acylcarnitine and six long-chain fatty acids, as well as methionine sulfoxide, a marker of oxidative stress, were accumulated in both CLDS1 and CLDS2. Acylcarnitines and fatty acids are metabolized via mitochondrial β-oxidation. Therefore, their accumulation suggests mitochondrial dysfunction in both CLDS1 and CLDS2 groups.

Depletion of NADP+ (related to NAD metabolism), gamma-tocopherol (a form of vitamin E), and DHEAS (androstenolone sulfate), as well as accumulation of ethanolamine, a phospholipid material, were also observed in both groups (Figure S5A). Although there was no significant difference in NAD+ levels in either group compared to the control (Figure S5A), NAD+ levels were highly correlated with NADP+ (Figure S5B), and fluctuations in NADP+ are linked to fluctuations in NAD+; it is thought that the decrease in NAD+, together with the increase in reactive oxygen species (ROS), contributes to mitochondrial dysfunction.^19^

The decrease in gamma-tocopherol suggests that ROS accumulation has an important role in pathogenicity in the liver. Previous studies have reported that gamma-tocopherol reduces liver injury, lipid peroxidation, and inflammation in lipopolysaccharide-induced NASH mouse models.^20^ In vitro studies have also reported that gamma-tocopherol, like alpha-tocopherol, has an inhibitory effect on oxidative stress.^21^ In our analysis, no significant difference was found for alpha-tocopherol, so there may be a specific effect of gamma-tocopherol. Gamma-tocopherol not only decreases ROS in Alzheimer’s disease, but also improves mitochondrial function.^22^

Three long-chain fatty acids and phosphatidylcholine metabolism-related metabolites, such as choline, cytidine-phosphocholine -choline, and lysophosphatidylcholine (24:0) were included among the metabolites accumulated only in CLDS2. Abnormalities in phosphatidylcholine metabolism are known to alter membrane fluidity.^23^

DHEAS was found to be decreased in both groups. DHEAS is an intermediate in the synthesis of sex hormones such as estrogen and testosterone.^24^ It has been reported that abnormalities in sex hormone metabolism are involved in the pathogenesis of NAFLD^25^ and that several hormone intermediates are reduced at serum levels with progressive fibrosis in NAFLD.^26^

It has also been reported that treatment with dehydroepiandrosterone (DHEA) could suppress the progression of NASH in a high-fat/high-cholesterol diet NASH mouse model.^27^

### 3.6 Investigating the Risk of Recurrence and Survival Rate After Surgery

To investigate the risk of recurrence in CLDS1 and CLDS2, an analysis comparing Kaplan–Meier recurrence-free survival (RFS) was conducted. Strikingly, no discernible disparity in recurrence rates was observed between CLDS1 and CLDS2 (Figure 5D). These findings imply that disparities in gene expression and metabolite profiles between the two subtypes do not contribute to recurrence. Clinical data on the cases submitted for analysis are presented in Table 2.

**Table 2:** Clinical Information on Cases with Recurrence Testing within CLDS1 versus CLDS2 Subgroups^a^

	CLDS1 (*n* = 15)	CLDS2 (*n* = 11)	*P* value
Age, median (IQR)	73.2 (69.0–74.7)	77.4 (71.5–82.1)	0.058
BMI, median (IQR)	23.7 (21.7–25.0)	22.2 (20.3–27.3)	0.98
*NAS*			
Steatosis			
0 (%)	7 (46.7)	9 (81.8)	0.11
1 (%)	8 (53.3)	2 (18.2)	
2 (%)	0 (0)	0 (0)	
3 (%)	0 (0)	0 (0)	
Hepatocyte ballooning			
0 (%)	9 (60)	7 (63.6)	0.74
1 (%)	3 (15.0)	1 (9.1)	
2 (%)	3 (15.0)	3 (27.3)	
Lobular inflammation			
1 (%)	4 (26.7)	7 (63.6)	0.045
2 (%)	11 (73.3)	3 (27.3)	
3 (%)	0 (0)	1 (9.1)	
Fibrosis			
F1–2 (%)	8 (53.3)	6 (54.5)	1
F3–4 (%)	7 (46.7)	5 (45.5)	
Size [mm], median (IQR)	23 (17–28)	30 (23–35)	0.077
Tumor number, median (IQR)	1 (1–1)	1 (1–1)	0.46
TNM classification			
I (%)	4 (26.7)	2 (18.2)	0.84
II (%)	5 (33.3)	5 (45.5)	
III (%)	1 (6.7)	2 (18.2)	
IVA (%)	3 (20)	1 (9.1)	
IVB (%)	2 (13.3)	1 (9.1)	
Differentiation			
Well (%)	3 (20.0)	4 (36.4)	0.41
Mod (%)	12 (80.0)	7 (63.6)	
Poor (%)	0 (0)	0 (0)	
Hepatic vein invasion			
vv0 (%)	15 (100)	10 (90.9)	0.42
vv1 (%)	0 (0)	1 (9.1)	
Portal vein invasion			
vp0 (%)	14 (93.3)	11 (100)	1
vp1 (%)	1 (6.7)	0 (0)	
AFP, median (IQR)	4.2 (2.7–15.4)	5 (4–14.3)	0.45
AFP-L3%, median (IQR)	0.5 (0.5–7.5)	4.5 (0.5–7.05) (*n* = 9)	0.92
DCP, median (IQR)	29 (19–146)	26 (20–50)	0.92

a Abbreviations: BMI, body mass index; NAS, NAFLD activity score; AFP, α-fetoprotein; DCP, des-γ-carboxy prothrombin.

To investigate factors contributing to recurrence, Cox proportional hazards regression analysis was performed on the same patients. The results showed that tumor size and des-γ-carboxy prothrombin (DCP) were associated with the risk of recurrence (*p* < 0.05) (Table S7).

We also investigated whether these subtypes affected postsurgical survival and found no difference in survival either (Figure 5E).

### 3.7 Therapeutic Potential

DEGs in CLDS1 and CLDS2 compared to controls were classified into six categories: CLDS1 upregulation (CLDS1UP), CLDS1 downregulation (CLDS1DW), CLDS2 upregulation (CLDS2UP), CLDS2 downregulation (CLDS2DW), common upregulation (ComUP), and common downregulation (ComDW). The Gene Ontology classifications that characterize each group are as follows: CLDS1UP: extracellular matrix and inflammation; CLDS1DW: organic acid synthesis; CLDS2DW: inflammation and signaling to external stimuli; ComUP: immunity; and ComDW: injury and regeneration. No specific characteristics were identified for CLDS2UP (Figure S6). We further investigated which cell types were enriched in the two groups using xCell. This revealed that CLDS1 was enriched in cells that have previously been reported to be associated with inflammation in the liver, such as monocytes, plasmacytoid dendritic cell (pDC), and mast cells.^28^ On the other hand, CLDS2 was enriched only in hepatic stellate cells (HSC) (Figure S6B).

**Figure 6:**
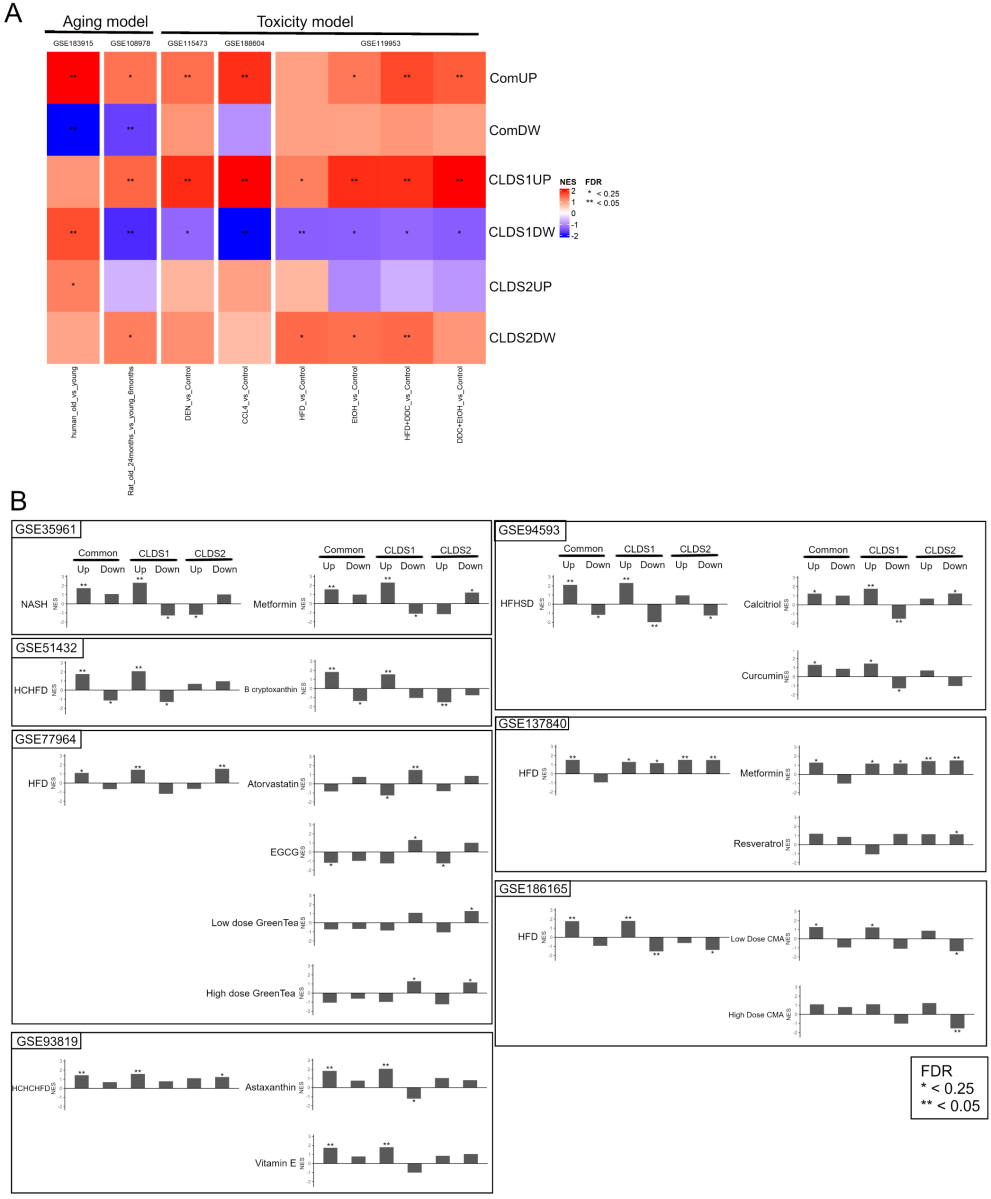
Signal changes in animal models. (A) Heatmap of signal changes due to factors inducing liver injury. (B) Treatment effectiveness investigation in the GEO data set. In each data set, the left-hand panel compares the control and high-fat diet in the GSEA analysis. The right-hand panel compares groups fed a high-fat diet and a high-fat diet with the respective additions in the GSEA analysis. The vertical axis represents the NES; * indicates FDR < 0.25, ** FDR < 0.05. HFD: high-fat diet; HCHFD: high-cholesterol and high-fat diet; HCHCHFD: high-cholesterol, high-cholate, and high-fat diet; HFHSD, high-fat, high-sugar diet.

We examined how expression of these genes could be altered by Diethylnitrosamine (DEN), 3,5-diethoxycarbonyl-1,4-dihydrocollidine, high-fat diet (HFD), ethanol (EtOH) and aging, which are factors known to induce liver injury, by performing GSEA against multiple data sets in the GEO database. We found that CLDS1UP and ComUP, except for GSE18395, were upregulated in multiple data sets regardless of the treatment (Figure 6A). In the Hyperlipidemic Animal model (GSE77964), controls treated with HFD were positively enriched in CLDS1UP and ComUP, but not when treated with green tea or epigallocatechin gallate (EGCG). In a NASH-induced model (GSE186165) using *Mesocricetus auratus*, HFD-treated controls were positively enriched in CLDS1UP and ComUP and negatively enriched in CLDS1DW, whereas treatment with metabolically active agents (CMA) (l-carnitine + NR + *N*-acetyl l-cysteine) showed volume-dependent improvement. Also, in a NASH-induced mouse model (GSE137840), HFD-treated controls were positively enriched in CLDS1UP, ComUP and CLDS2UP, which was improved by treatment with resveratrol (Figure 6B).

## 4 Discussion

Compared with the control group, acylcarnitines and fatty acids were accumulated in the CLD group, but unexpectedly, changes in amino acids were limited to a few types (Figure 1D). Amino acid imbalances have been frequently reported in CLD,^29,30^ but most are based on serum measurements. Measurements within the liver tissue may yield different profiles. In this study, the five amino acids for which significant differences were found (valine, leucine, isoleucine, lysine, and tyrosine) are known to be metabolized in the mitochondria in the liver,^31^ supporting mitochondrial dysfunction associated with acylcarnitine and fatty acid accumulation.

Metabolome analysis based on background liver differences showed only a few varied metabolites among age, DM, and HL. Further investigation is required to understand the association between these metabolites and disease. Decreased serum DHEAS is reported in association with DM in men.^32^ DM is a known risk factor for HCC, and reduced DHEAS may be a contributing factor. As fibrosis progressed, fatty acids decreased, and metabolites related to glycolysis and the pentose phosphate pathway accumulated. Patients with congenital transaldolase deficiency develop liver cirrhosis in infancy, supporting the role of the pentose phosphate pathway in liver cirrhosis.^33^

As proposed by the multihit hypothesis, the pathogenesis of CLD is considered to be a complex mechanism.^34^ Although many previous studies have attempted to elucidate the mechanism of carcinogenesis, various mechanisms have been proposed, and some have not yielded consistent results. To elucidate the pathogenesis in more detail, it is necessary to eliminate heterogeneity within a cohort. MOVICS-based classification was used to partition the patients into more homogeneous groups that were found to differ with respect to age and inflammation stage (Figure 3). In comparison with PLS/PLS-NAFLD, there was a tendency for CLDS1 to have more poor/high-risk cases. This result is reasonable because genes related to inflammation are enriched in the poor/high-risk group (Figure S4). The reason why RFS was not significantly different from CLDS2 in this study, despite the large number of PLS-poor prognosis cases in CLDS1 is due to the small number of cases and the lack of a one-to-one correspondence between CLDS and PLS. The possibility of metastatic recurrence, as opposed to de novo carcinogenesis, cannot be completely ruled out in our cohort of recurrent cases. Similar to RFS, postsurgical survival analysis showed no difference between the two groups (Figure 5E). This is likely due to differences in treatment options at the time of recurrence based on age and performance status, as well as the inherently higher risk of mortality in the elderly group for various reasons.

GSEA revealed that CLDS1 is enriched in EMT, inflammation, and NF-κB signaling, which have been reported in many studies as oncogenic mechanisms and could reflect differences with respect to inflammation grade. Excessive PARP activation contributes to NF-κB signaling,^35^ and upregulation of *PARP1* was identified only in CLDS1 (Figure S5C). Therefore, NF-κB activation in CLDS1 may induce fibrosis via EMT or inflammatory signaling through *PARP1* upregulation. *PARP1* may be a promising therapeutic target for CLDS1, as it has been reported that PARP inhibitors improve NAFLD in a mouse model.^36^

Metabolome analysis confirmed that NADP+ was decreased in both CLDS1 and CLDS2 compared to controls. Recent studies have also provided increasing evidence of the importance of NAD+ in NAFLD.^37,38^ In the present study, NAD+ showed a decreasing but nonsignificant trend, and a high correlation was observed between NADP+ and NAD+ (Figure S5B). *NAMPT*, the rate-limiting enzyme for NAD+ synthesis, fluctuated in the opposite direction between CLDS1 and CLDS2, suggesting different causes for the decline in NADP+ (Figure S5C). In CLDS1, where *NAMPT* is up-regulated, the supply of NAD+ is thought to be sufficient, but it is inferred that consumption is even higher. This may be related to the upregulation of *PARP1* described above. On the other hand, in CLDS2, when *NAMPT* is downregulated, the supply of NAD+ is not expected to be sufficient. It has been reported that when NAD+ is decreased, the activity of sirtuin, a NAD-consuming enzyme, decreases, leading to mitochondrial dysfunction.^37^ NADP+ has also been shown to be an important metabolic product that characterizes CLD through multiomics analysis using mixOmics (Figure S3B). Acylcarnitine, a metabolic intermediate of β-oxidation, was found to be accumulated in CLDS1, and fatty acids were found to be accumulated in CLDS2.

Fujiwara et al. reported that acylcarnitine accumulated in HCC tissues of mice treated with a high-fat diet plus DEN in a study using a NASH mouse model and that carnitine palmitoyltransferase 2 (*CPT2*), which converts acylcarnitine to acyl-CoA, was downregulated.^39^ The accumulation of acylcarnitines are observed not only in tumor tissue but also in adjacent nontumor tissue, suggesting that they play an important role in NASH-associated HCC. Our RNA-Seq results showed no change in *CPT2* (data not shown). It has recently been reported that inactivation of sirtuin 3 (*SIRT3*) in platelets results in increased acetylation of K79 in *CPT2* and accumulation of acylcarnitines.^40^ As NAD+ (NADP+) depletion was also observed at the same time in this study, it may be necessary to consider abnormal acetylation of *CPT2* as one of the mechanisms for the abnormalities in β-oxidation identified in CLD.

In contrast to CLDS1, carcinogenesis and chronic liver disease in CLDS2 cannot be explained by these mechanisms. Metabolome analysis suggests instead that dysregulation of phosphatidylcholine synthesis plays a role in carcinogenesis in CLDS2. Kawamura et al. found that liver-specific PTEN/SCAP double knockout mice acquire a “burned-out NASH″-like phenotype.^41^ They concluded that changes in phosphatidylcholine composition due to decreased expression of lysophosphatidylcholine acyltransferase 3 (*LPCAT3*) was associated with a decrease in phosphatidylcholine synthesis, endoplasmic reticulum (ER) stress, and hepatocellular damage.^41^ In the current study, although there was no significant difference, the gene expression level of *LPCAT3* was lower in CLDS2 compared to the control group (*p* = 0.09) (Figure S5C). Changes in phosphatidylcholine composition have also been reported to be caused by cellular senescence.^42^

Since CLDS2 is characterized by older age, it is not surprising that age-induced changes are more pronounced. *TP53*, *CDKN1A*, *CDKN2A*, and *LMNB1* are often used as indicators of aging, but they have been found to be inconsistent across organs and cells,^43^ so caution is needed in interpretation. We examined expression of these genes and found the following: (i) *TP53* was not obviously changed, (ii) *CDKN1A* was upregulated only in CLDS1, (iii) *CDKN2A* was upregulated in both groups, and (iv) *LMNB1* was upregulated in CLDS1 and downregulated in CLDS2 (Figure S5C). Wei et al. reported that LNMB1 is reduced and nuclear morphology is altered in some young NAFLD patients with severe steatosis.^44^

It has been reported that cellular senescence results in the Senescence-Associated Secretory Phenotype (SASP), which in turn releases inflammatory cytokines and other factors.^45^ However, if CLDS2 has acquired SASP, it would be contradictory that inflammation-related signals are negatively enriched in CLDS2. Nacarelli et al. reported that SASP is regulated by NAD metabolism and that increased expression of *NAMPT* promotes the secretion of inflammatory cytokines.^46^ This is consistent with our findings of low levels of inflammation in CLDS2 with reduced expression of *NAMPT*. They also reported that *NAMPT* expression is upregulated in oncogene-induced senescence but not in replicative senescence. This evidence may help to explain the difference between CLDS1 and CLDS2.

It is also interesting that cholesterol homeostasis was negatively enriched in CLDS2 in GSEA. This is because abnormalities in cholesterol homeostasis have been reported to be associated with carcinogenesis in HCC.^47^ In addition, abnormalities in sex hormones produced from cholesterol have been reported to be involved in NAFLD.^27,48^ In our study, there was no significant difference in cholesterol by metabolome analysis, and unfortunately, sex hormones were not detected, but the intermediate DHEAS was found to be decreased both in CLDS1 and CLDS2 (Figure S5A). DHEAS is the most abundant of the circulating steroids and is produced from DHEA in the adrenal glands and liver.^49^ Gene expression levels of *HSD17B14*, *CYP3A7*, *CYP1A1*, *CYP2C19* and *CYP3A4* involved in sex hormone metabolism were significantly altered (Figure S7A). These may contribute to the progression of NAFLD by dramatically altering metabolic processes.^50−52^ DHEAS and *CYP2C19* were also identified as important factors characterizing CLD in the mixOmics analysis and may be involved in the pathology independent of the subtype. However, DHEAS has been shown to decrease with age,^53^ so whether it is involved in carcinogenesis needs further investigation. We also found that CLDS2 is more enriched in HSC by cell enrichment analysis (Figure S6B). Wahid et al. report that aging modulates immune responses, impairs regenerative capacity via HSC activation, affects adipokines and cholesterol levels, and increases susceptibility to liver fibrosis in a rat model.^54^ This is consistent with our result and suggests that HSCs may play a key role in CLDS2 carcinogenesis.

Several inflammatory markers have been reported to be associated with RFS.^55^ As CLDS1 is associated with a higher inflammatory stage than CLDS2 (Figure 3), it was thought that there would be a difference in RFS, but surprisingly this was not the case. This suggests that commonly up- or down-regulated genes or accumulated or depleted metabolites are more important for recurrence compared to the control group. Commonly up-regulated genes include those reported to be associated with HCC, such as *AKR1B10*(56) and *SPP1*(57) (Figure S7B). However, Cox proportional hazards regression analysis showed that tumor size and DCP were associated with recurrence. The fact that a larger tumor was involved in the recurrence suggests that the tumor was not completely removed during surgery and may have metastasized and recurred.

We investigated the various data sets registered in public databases and revealed that the genes upregulated only in CLDS1 and those commonly upregulated in CLDS1 and CLDS2 were similarly altered in NASH/NAFLD animal models. These signals varied widely depending on the treatment, but improvement was observed in models treated with EGCG, metabolically active agents (l-carnitine + NR + *N*-acetyl l-cysteine), and resveratrol, which may hold promise as a treatment to restore the molecular abnormalities we have identified that are associated with CLD progression. We believe that this treatment may be expected to restore the molecular abnormalities associated with the progression of CLD that we have identified in this study. However, no improvement was observed in models treated with vitamin E. This was contrary to our expectation, as gamma-tocopherol, a form of vitamin E, was decreased in both groups in our analysis.

Although various therapeutic strategies have been explored,^38^ no effective treatment for NASH/NAFLD has yet been established. The multiomics analysis of the transcriptome and metabolome in this study may provide useful information for future therapeutic strategies.

Collectively, we identified two CLD patient subgroups that differed with respect to carcinogenic mechanism, one characterized by a high inflammatory stage and the other comprised mainly of elderly patients. Elevated inflammatory signals were observed from RNA-Seq in the inflammation-related group. Furthermore, abnormalities in β-oxidation and NAD metabolic pathways, as well as depletion of DHEAS and gamma-tocopherol, were identified in both groups. It is suggested that these metabolic abnormalities may be directly involved in carcinogenesis, particularly in the age-related group. This underscores the significance of heterogeneity in molecular abnormalities in CLD and the selection of therapeutic targets.

The findings of this study suggest the necessity of cancer prevention strategies tailored to the contexts of inflammation and aging. While the mechanisms of aging-related carcinogenesis could have been further elucidated, this remains a subject for future investigation.

## 5 Limitations

One limitation of this study is that not all patients were tested for AIH markers, specifically ASMA and antiliver kidney microsome antibodies (anti-LKM antibodies).

## Supporting Information Available

RNA-Seq and metabolome analysis flowchart (Figure S1); optimal cluster number of MOVICS (Figure S2); biomarker discovery using mixOmics (Figure S3); association between the prognostic liver signature (PLS) and NAFLD-PLS (Figure S4); significant differences in metabolites and RNA between groups (Figure S5); annotations by Gene Ontology for groups of genes with variation in CLDS1 and CLDS2 (Figure S6); genes involved in steroid metabolism and closely associated with carcinogenesis (Figure S7); details of basic scan and dual scan (Text S1) (PDF)
Quantification and quality checks from total RNA preparation to sequencing (Table S1); information on the material used for metabolome analysis (Table S2); results of differential gene expression analysis between control and CLD (Table S3); results of differential metabolites analysis between control and CLD (Table S4); comparison of metabolomes with different backgrounds (Table S5); results of GSEA analysis of control versus each subgroup (Table S6); search for factors contributing to recurrence using Cox proportional hazards regression analysis (Table S7) (XLSX)

## Abbreviations

AC: acylcarnitine
ALT: alanine transaminase
AST: aspartate transaminase
CLD: chronic liver disease
DEG: differentially expressed gene
DEM: differential metabolites
DHEA: dehydroepiandrosterone
DHEAS: dehydroisoandrosterone-3-sulfate-1
DM: diabetes mellitus
EMT: epithelial–mesenchymal transition
FA: fatty acid
γGTP: gamma-glutamyl transpeptidase
GEO: Gene Expression Omnibus
GSEA: Gene Set Enrichment Analysis
HCC: hepatocellular carcinoma
HFD: high-fat diet
LPCAT3: lysophosphatidylcholine acyltransferase 3
MAD: median absolute deviation
MOVICS: Multi-Omics Integration and VIsualization in Cancer Subtyping
NAD: nicotinamide adenine dinucleotide
NADP: nicotinamide adenine dinucleotide phosphate
NAFLD: nonalcoholic fatty liver disease
NAMPT: nicotinamide phosphoribosyltransferase
NAS: NAFLD Activity Score
NASH: nonalcoholic steatohepatitis
PCA: principal component analysis
PLS: prognostic liver signature
RFS: relapse-free survival
ROS: reactive oxygen species
