# Supplementary material for: Multiomics Analysis of Liver Molecular Dysregulation Leading to Nonviral-Related Hepatocellular Carcinoma Development
Source: J Proteome Res. 2025 Feb 21;24(3):1102–17. doi: 10.1021/acs.jproteome.4c00729 (PMC11894656; doi:10.1021/acs.jproteome.4c00729)
Supplement: Supplementary file 1 — pr4c00729_si_001.pdf [file pr4c00729_si_001.pdf]

Supporting information

Multi-omics analysis of liver molecular dysregulation leading to non-viral related hepatocellular carcinoma development

Hikaru Nakahara[1,2], Atsushi Ono[1], C. Nelson Hayes[1], Yuki Shirane[1], Ryoichi Miura[1], Yasutoshi Fujii[1,3], Yosuke Tamura[1], Shinsuke Uchikawa[1], Hatsue Fujino[1], Takashi Nakahara[4], Eisuke Murakami[1], Masami Yamauchi[5], Tomokazu Kawaoka[1], Daiki Miki[1], Masataka Tsuge[1,6], Tsuyoshi Kobayashi[7], Hideki Ohdan[7], Koji Arihiro[8], Shiro Oka[1]*

1 Department of Gastroenterology, Graduate School of Biomedical & Health Sciences, Hiroshima University, Hiroshima 734-8551, Japan.

2 Department of Clinical and Molecular Genetics, Hiroshima University, Hiroshima 734-8551, Japan.

3 Department of Clinical Oncology, Graduate School of Biomedical and Health Sciences, Hiroshima University, Hiroshima 734-8551, Japan.

4 Hiroshima Prefectural Hospital Gastroenterology & Hepatology, Hiroshima 734-8530, Japan

5 Hiroshima Prefectural Hospital, Department of Clinical Oncology, Hiroshima 734-8530, Japan

6 Research Center for Hepatology and Gastroenterology, Hiroshima University, Hiroshima 734-8551, Japan

7 Department of Gastroenterological and Transplant Surgery, Graduate School of Biomedical and Health Sciences, Hiroshima University, Hiroshima 734-8551, Japan.

8 Department of Anatomical Pathology, Hiroshima University Hospital, Hiroshima 734-8551, Japan.

Table of contents

Fig. S1. RNA-Seq and metabolome analysis flowchart

Fig. S2. Optimal cluster number of MOVICS

Fig. S3. Biomarker discovery using mixOmics

Fig. S4. Association between the Prognostic liver signature (PLS) and NAFLD-PLS

Fig. S5. Significant differences in metabolites and RNA between groups

Fig. S6. Annotations by Gene Ontology for groups of genes with variation in CLDS1 and CLDS2

Fig. S7. Genes involved in steroid metabolism and closely associated with carcinogenesis

Table S1. Quantification and quality checks from total RNA preparation to sequencing

Table S2. Information on the material used for metabolome analysis

Table S3. Results of differential gene expression analysis between control and CLD

Table S4. Results of differential metabolites analysis between control and CLD

Table S5. Comparison of metabolomes with different backgrounds

Table S6. Results of GSEA analysis of control versus each subgroup

Table S7. Search for factors contributing to recurrence using Cox proportional hazards regression analysis

Text S1. Details of Basic Scan and Dual Scan

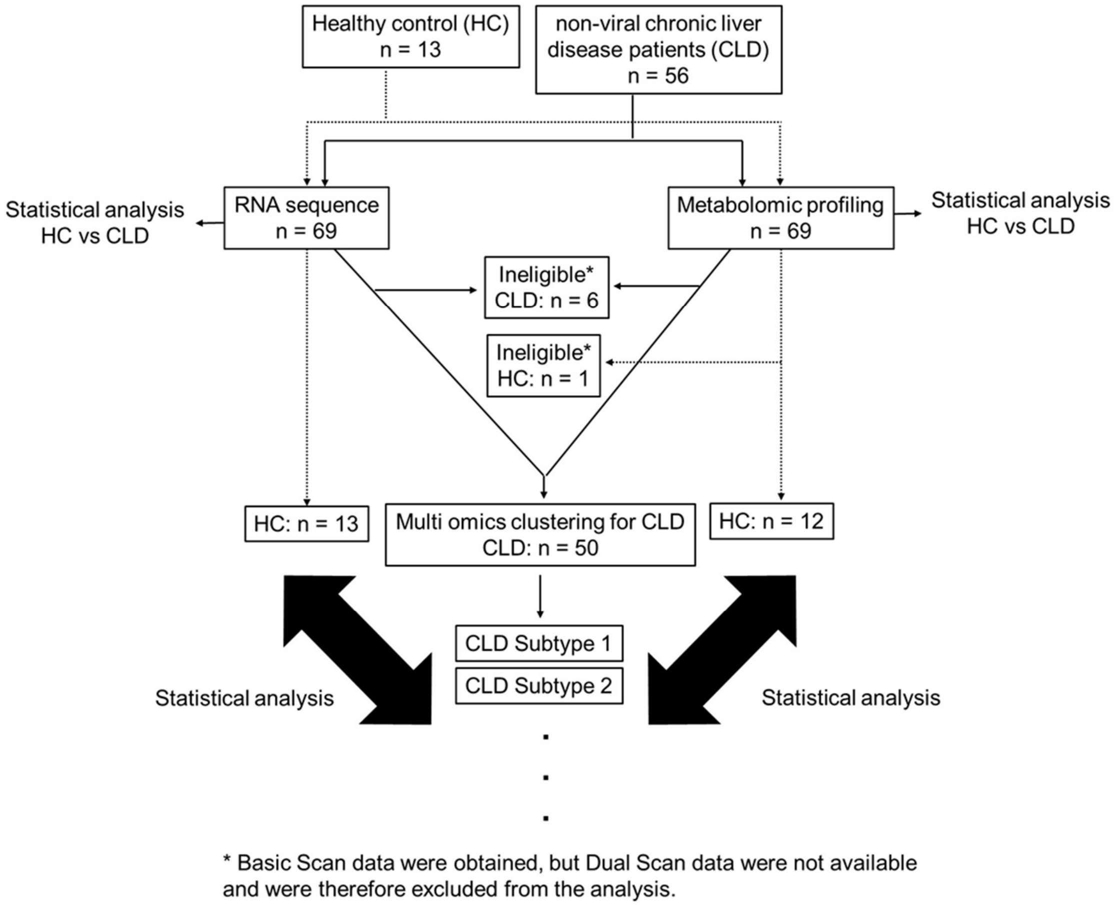

Fig. S1 RNA-Seq and metabolome analysis flowchart. Healthy control and chronic liver disease groups were compared, followed by subtyping to remove heterogeneity.

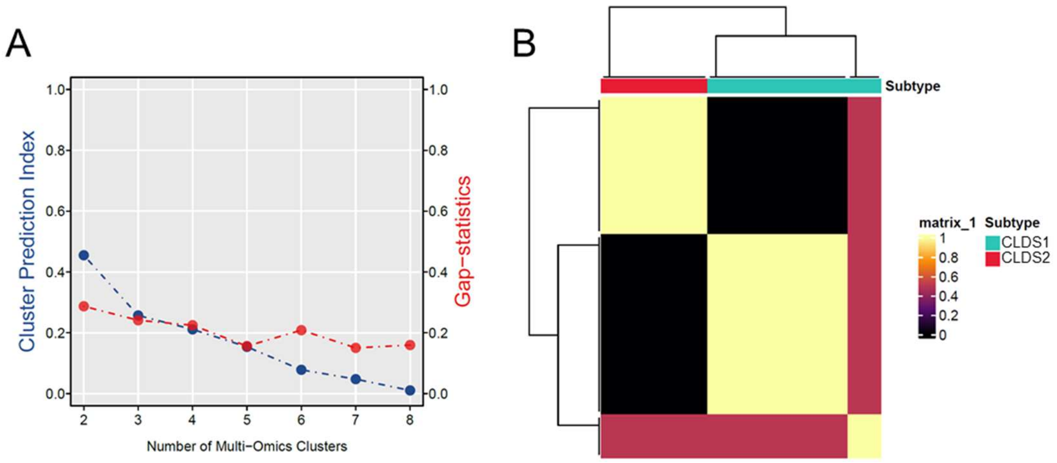

Fig.S2 (A) Identification of optimal cluster number by calculating clustering prediction index (blue line) and Gaps-statistics (red line). (B) Consensus heatmap based on results from 2 multi-omics integrative clustering algorithms, IntNMF and iClusterBayes with a cluster number of 2.

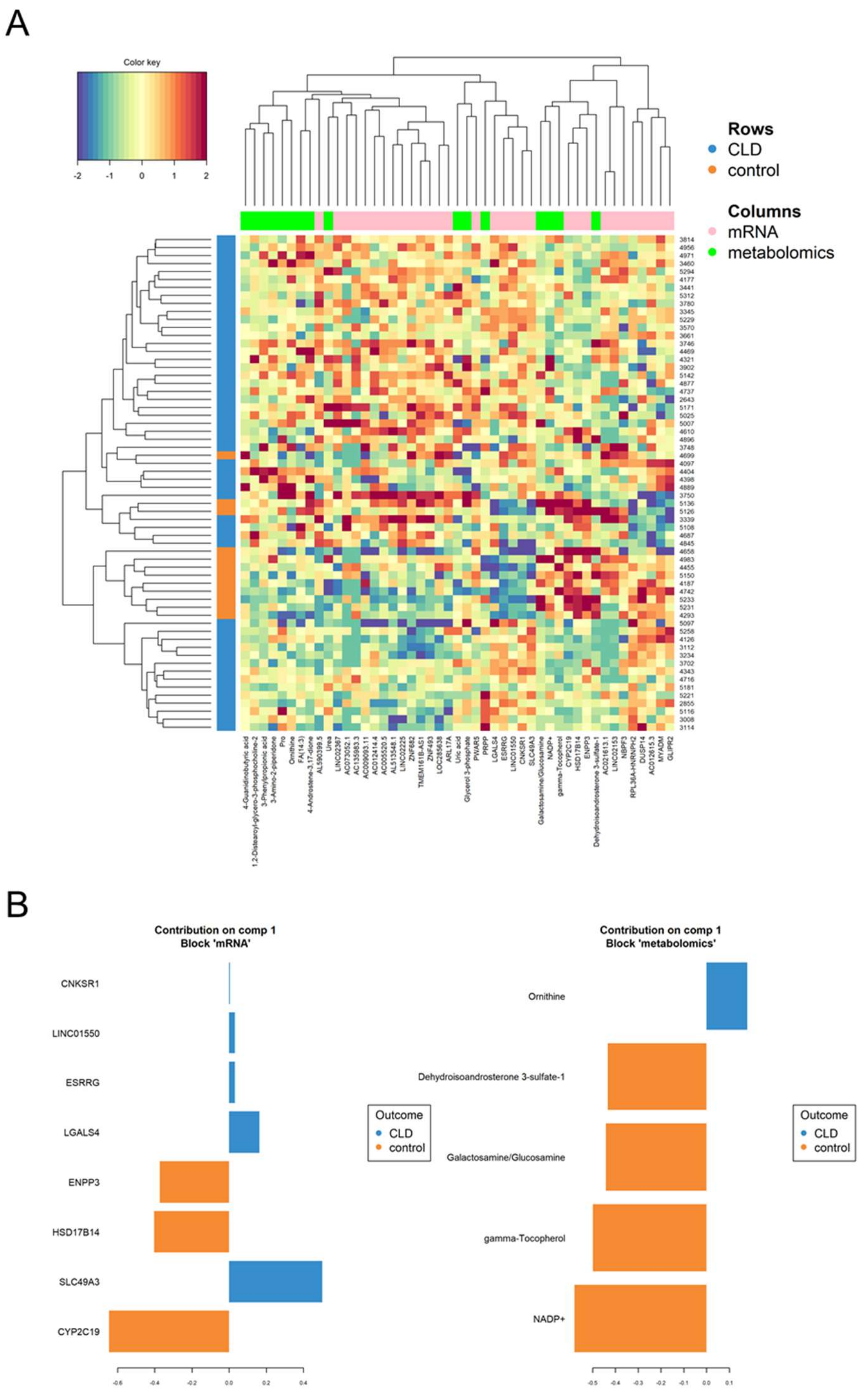

Fig.S3 Multi-omics analysis using mixOmics. **(**A) Heatmap showing relationships between gene and metabolite profiles with respect to control and CLD groups. (B) Loading plot of factors that contribute most to discriminating between control and CLD groups.

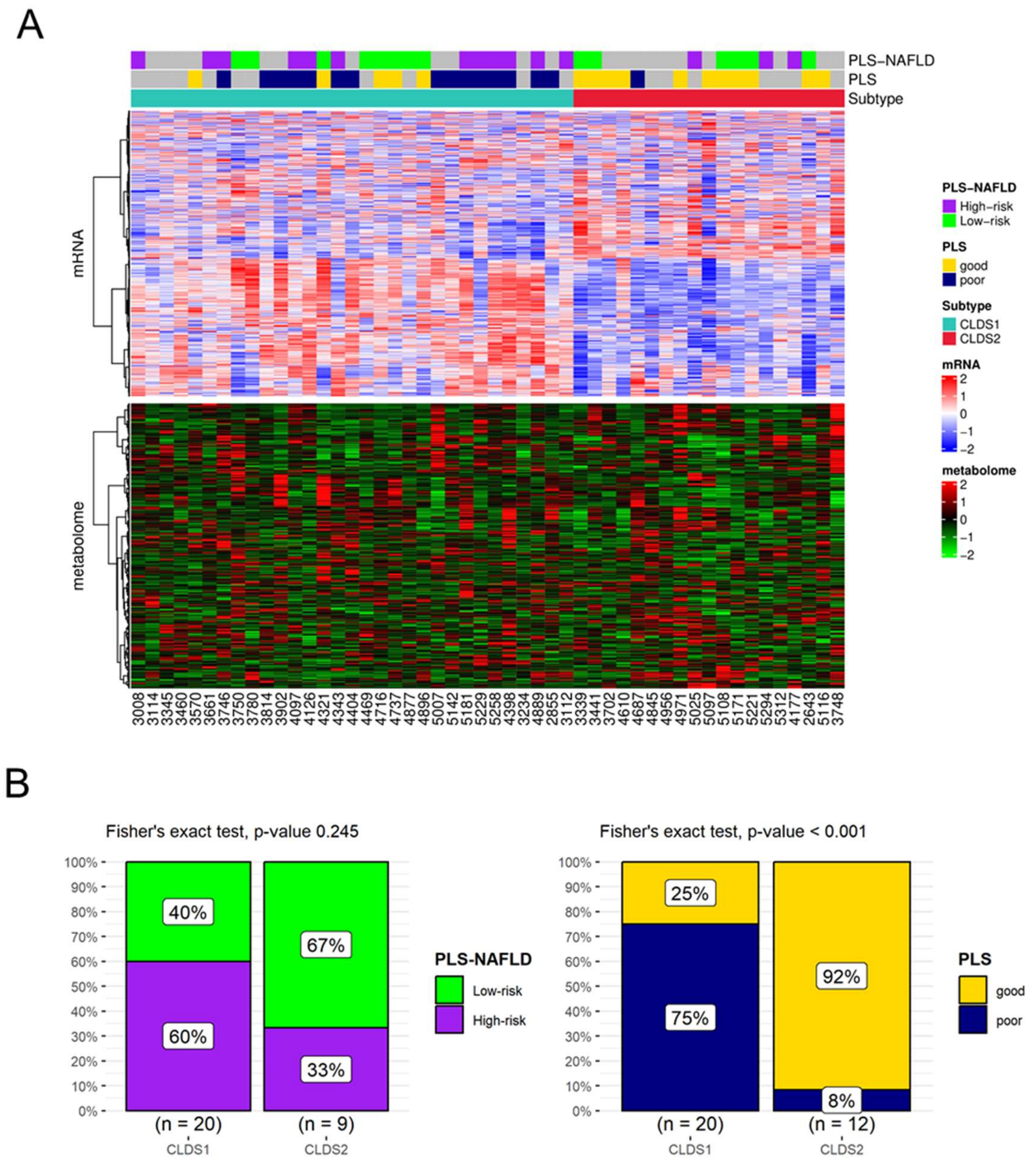

Fig. S4 (A) Association between the Prognostic liver signature (PLS) and NAFLD-PLS of Hoshida et al. The significance level was set as FDR < 0.05, and those that did not meet the significance level were set as NA (grey color). (B) Percentages of PLS/PLS-NAFLD classes in each subtype and results of the Fisher exact test.

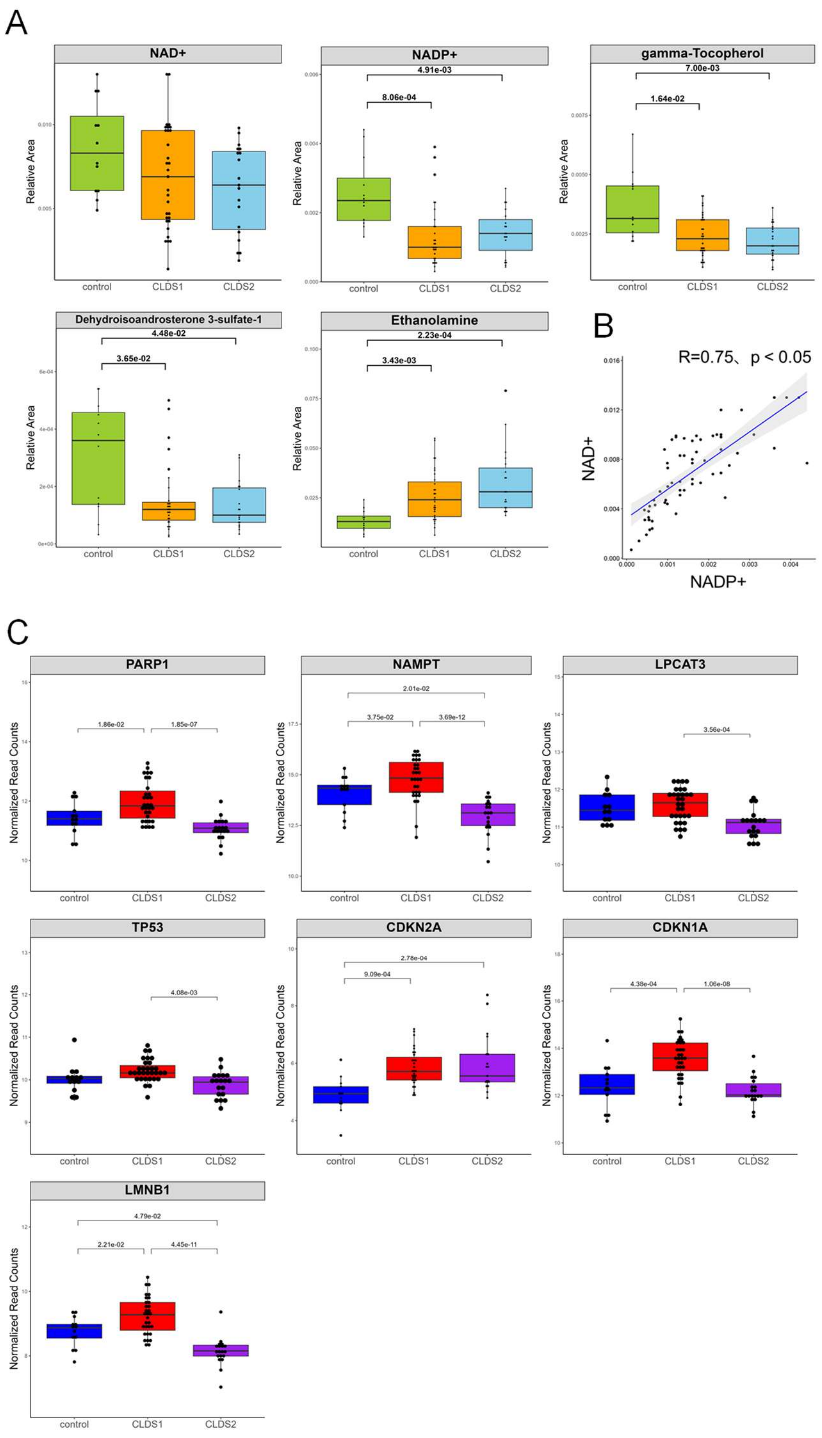

Fig. S5 (A) Boxplots of NAD+ and NADP+. (B) Pearson correlation analysis of NAD+ and NADP+. (C) Genes related to NAD+ metabolism, membrane phospholipids, and cellular senescence.

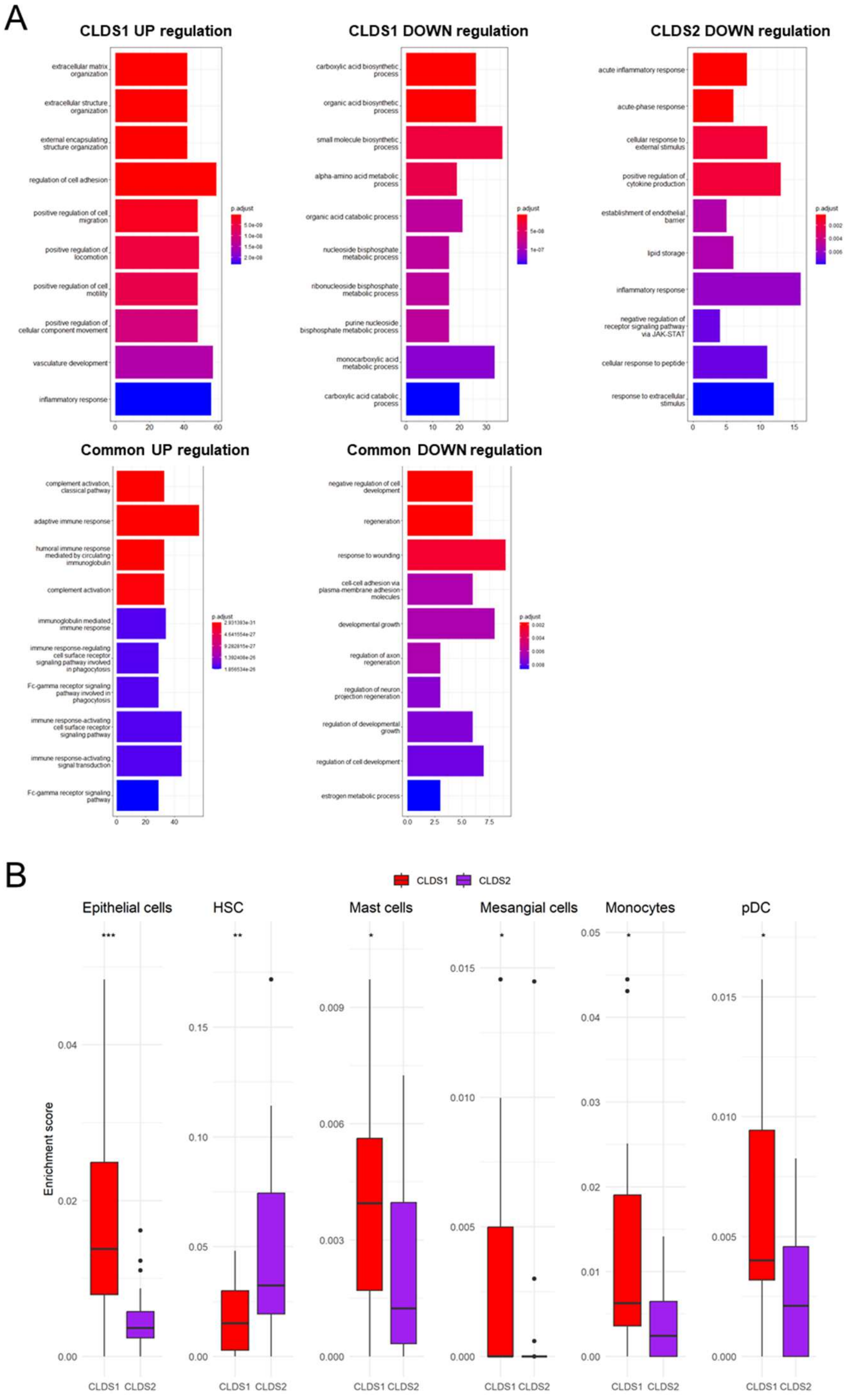

Fig. S6 (A) Annotations by Gene Ontology for groups of genes with variation in CLDS1 and CLDS2. (B) Cell enrichment score plot for CLDS1 and CLDS2. *p < 0.05, **p < 0.01, ***p < 0.001.

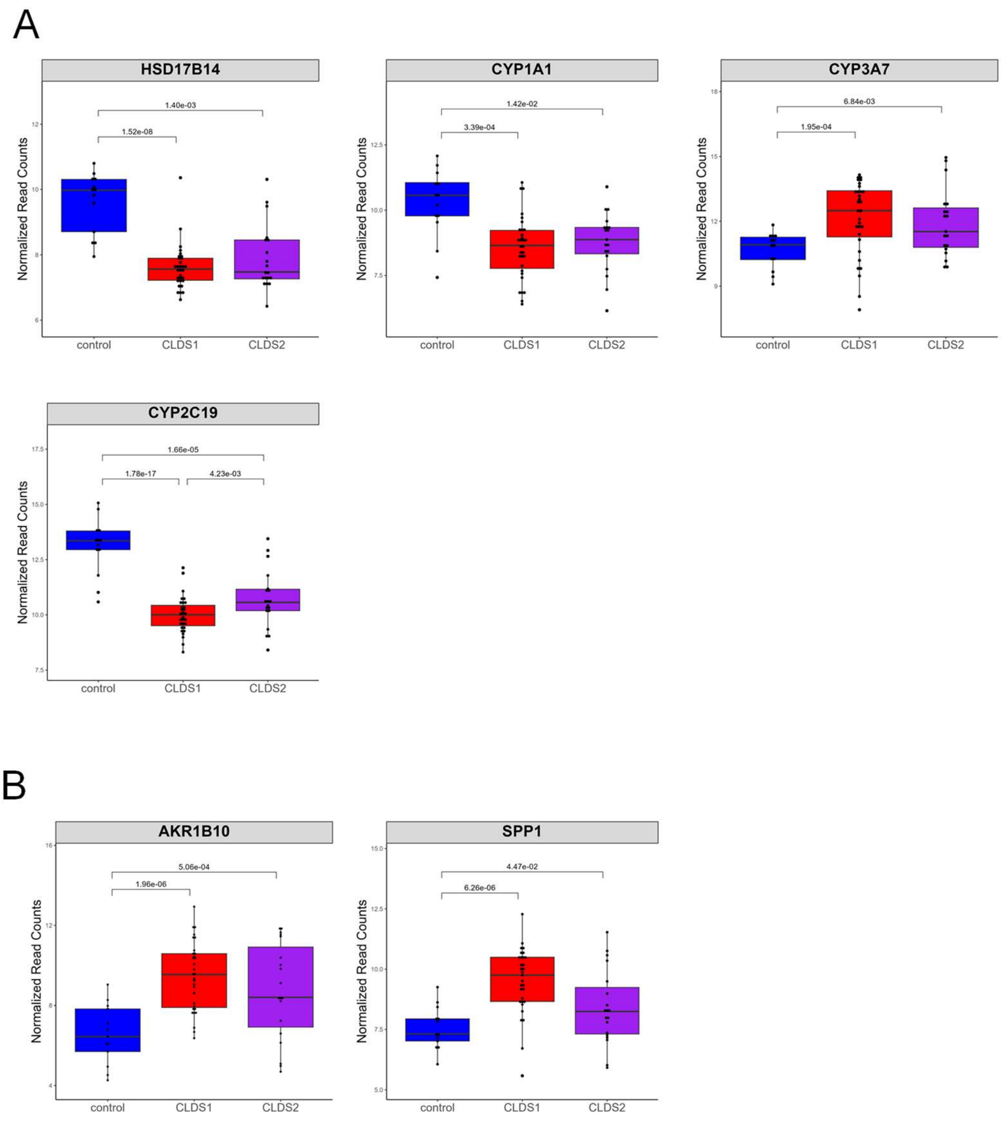

Fig. S7 (A) Genes involved in steroid metabolism that were variable in CLDS1 and CLDS2. (B) Genes commonly up-regulated in CLDS1 and CLDS2 that are closely associated with carcinogenesis.

Text S1 Details of Basic Scan and Dual Scan

Metabolome analysis (Basic Scan)

Metabolome analysis was performed following the Basic Scan protocol provided by HMT. Capillary electrophoresis time-of-flight mass spectrometry (CE-TOFMS) was employed, utilizing methodologies previously reported in the literature[1, 2]. In this process, CE-TOFMS measurements were conducted using an Agilent CE system paired with an Agilent 6210 TOF mass spectrometer (Agilent Technologies, Santa Clara, CA, USA). Instrument operations were managed using Agilent G2201AA ChemStation software (version B.03.01) and connected via a fused silica capillary (50 μm inner diameter, 80 cm total length). Commercially available electrophoresis buffers (H3301-1001 for cation analysis and I3302-1023 for anion analysis, HMT) served as the electrolytes. Mass spectrometry data were acquired in the range of m/z 50–1,000. Peak extraction, including the determination of m/z values, peak areas, and migration times (MTs), was performed using MasterHands, a dedicated software tool for automated data integration developed at Keio University (Tsuruoka, Yamagata, Japan)[3]. Peaks identified as isotopomers, adduct ions, or by-products of known metabolites were excluded, and the remaining peaks were annotated using HMT's metabolite database based on their m/z values and migration times. Peak areas were subsequently normalized relative to internal standards and sample quantities, providing relative metabolite levels. Additionally, absolute quantification of 110 primary metabolites was achieved using one-point calibration with standard compounds for each target metabolite.

Metabolome analysis (Dual Scan)

Metabolome analysis was performed using HMT's Dual Scan package, which combines capillary electrophoresis time-of-flight mass spectrometry (CE-TOFMS) and liquid chromatography time-of-flight mass spectrometry (LC-TOFMS). The procedures followed were adapted from previously established methods[1, 2]. For CE-TOFMS analysis, an Agilent capillary electrophoresis system was utilized in conjunction with an Agilent 6210 time-of-flight mass spectrometer (Agilent Technologies, Santa Clara, CA, USA). LC-TOFMS analysis was conducted using an Agilent 1200 high-performance liquid chromatography (HPLC) pump paired with the same model of mass spectrometer. CE system operations were managed through Agilent G2201AA ChemStation software

(version B.03.01), while LC analysis employed MassHunter software (Agilent Technologies).Mass spectrometry scans covered the m/z range of 50–1,000. Data processing, including the extraction of m/z values, peak areas, and migration times (MTs) for CE-TOFMS or retention times (RTs) for LC-TOFMS, was performed using MasterHands, an automated integration tool developed at Keio University (Tsuruoka, Yamagata, Japan)[3]. Peaks corresponding to isotopomers, adduct ions, or by-products of known metabolites were filtered out, while the remaining signals were annotated using HMT's metabolite database by comparing m/z values with MTs or RTs. Peak areas were normalized against internal standards and sample amounts to calculate relative metabolite levels. Additionally, absolute quantification of 110 key metabolites was carried out using one-point calibrations with their respective standard compounds.

REFERENCES

(1) Ohashi, Y.; Hirayama, A.; Ishikawa, T.; Nakamura, S.; Shimizu, K.; Ueno, Y.; Tomita, M.; Soga, T. Depiction of metabolome changes in histidine-starved Escherichia coli by CE-TOFMS. *Mol Biosyst* **2008**, *4* (2), 135-147. DOI: 10.1039/b714176a From NLM Medline.

(2) Ooga, T.; Sato, H.; Nagashima, A.; Sasaki, K.; Tomita, M.; Soga, T.; Ohashi, Y. Metabolomic anatomy of an animal model revealing homeostatic imbalances in dyslipidaemia. *Mol Biosyst* **2011**, *7* (4), 1217-1223. DOI: 10.1039/c0mb00141d From NLM Medline.

(3) Sugimoto, M.; Wong, D. T.; Hirayama, A.; Soga, T.; Tomita, M. Capillary electrophoresis mass spectrometry-based saliva metabolomics identified oral, breast and pancreatic cancer-specific profiles. *Metabolomics* **2010**, *6* (1), 78-95. DOI: 10.1007/s11306-009-0178-y From NLM PubMed-not-MEDLINE.
